# Brain tumor segmentation using neuro-technology enabled intelligence-cascaded U-Net model

**DOI:** 10.3389/fncom.2024.1391025

**Published:** 2024-04-03

**Authors:** Haewon Byeon, Mohannad Al-Kubaisi, Ashit Kumar Dutta, Faisal Alghayadh, Mukesh Soni, Manisha Bhende, Venkata Chunduri, K. Suresh Babu, Rubal Jeet

**Affiliations:** ^1^Department of Digital Anti-Aging Healthcare, Inje University, Gimhae, Republic of Korea; ^2^Department of Computer Science, Al-Maarif University College, Al-Anbar Governorate, Iraq; ^3^Department of Computer Science and Information Systems, College of Applied Sciences, AlMaarefa University, Riyadh, Saudi Arabia; ^4^Department of Computer Science and Information Systems, College of Applied Sciences, AlMaarefa University, Riyadh, Saudi Arabia; ^5^Department of CSE, University Centre for Research and Development, Chandigarh University, Mohali, Punjab, India; ^6^Dr. D. Y. Patil Vidyapeeth, Pune, Dr. D. Y. Patil School of Science & Technology, Tathawade, Pune, India; ^7^Department of Mathematics and Computer Science, Indiana State University, Terre Haute, IN, United States; ^8^Department of Biochemistry, Symbiosis Medical College for Women, Symbiosis International (Deemed University), Pune, India; ^9^Chandigarh Engineering College, Jhanjeri, Mohali, India

**Keywords:** brain tumours, image segmentation, deep learning, expectation maximization, convolutional neural networks (CNNs), magnetic resonance imaging (MRI)

## Abstract

According to experts in neurology, brain tumours pose a serious risk to human health. The clinical identification and treatment of brain tumours rely heavily on accurate segmentation. The varied sizes, forms, and locations of brain tumours make accurate automated segmentation a formidable obstacle in the field of neuroscience. U-Net, with its computational intelligence and concise design, has lately been the go-to model for fixing medical picture segmentation issues. Problems with restricted local receptive fields, lost spatial information, and inadequate contextual information are still plaguing artificial intelligence. A convolutional neural network (CNN) and a Mel-spectrogram are the basis of this cough recognition technique. First, we combine the voice in a variety of intricate settings and improve the audio data. After that, we preprocess the data to make sure its length is consistent and create a Mel-spectrogram out of it. A novel model for brain tumor segmentation (BTS), Intelligence Cascade U-Net (ICU-Net), is proposed to address these issues. It is built on dynamic convolution and uses a non-local attention mechanism. In order to reconstruct more detailed spatial information on brain tumours, the principal design is a two-stage cascade of 3DU-Net. The paper’s objective is to identify the best learnable parameters that will maximize the likelihood of the data. After the network’s ability to gather long-distance dependencies for AI, Expectation–Maximization is applied to the cascade network’s lateral connections, enabling it to leverage contextual data more effectively. Lastly, to enhance the network’s ability to capture local characteristics, dynamic convolutions with local adaptive capabilities are used in place of the cascade network’s standard convolutions. We compared our results to those of other typical methods and ran extensive testing utilising the publicly available BraTS 2019/2020 datasets. The suggested method performs well on tasks involving BTS, according to the experimental data. The Dice scores for tumor core (TC), complete tumor, and enhanced tumor segmentation BraTS 2019/2020 validation sets are 0.897/0.903, 0.826/0.828, and 0.781/0.786, respectively, indicating high performance in BTS.

## 1 Introduction

There are two main types of brain tumours, benign and malignant, which are defined as aberrant cell growths in the brain (Anselmi and Patel, 2022). Any kind of brain tumour, benign or malignant, poses a serious risk to human health because it presses on other parts of the skull and damages the brain tissue around (Bianchetti et al., 2021). The importance of early identification in the treatment of brain tumours has been demonstrated in clinical practice. The gold standard for brain tumor detection right now is magnetic resonance imaging (MRI), which can produce a variety of tissue contrast pictures that help doctors diagnose and treat the disease. Precise segmentation is essential for the clinical diagnosis and management of brain tumours. Accurate automated segmentation of brain tumours is a significant challenge in the field of neuroscience due to their diverse sizes, shapes, and locations. Recently, U-Net has become the model of choice for resolving medical picture segmentation problems because to its computational intelligence and succinct architecture. There is an immediate need for computer-assisted automated BTS since manually segmenting and analysing brain tumours in MRI images is a tedious and time-consuming process (Dhiman et al., 2023). The uneven and unclear borders, different sizes, and forms of brain tumours make automated high-precision BTS a tough job in medical image analysis at present.

BTS tasks have seen the effective use of deep learning in recent years, leading to its progressive mainstreaming in the area. Fully convolutional networks (FCNs) were built in 2014 by Abdar et al. (2021) substituting convolutional layers for fully connected ones in convolutional neural networks (CNNs) (Ahmed and Prakasam, 2023). This solved the spatial discontinuity problem in image segmentation and allowed for successful pixel-level image classification, which laid the groundwork for deep learning image segmentation (Poulakis and Westman, 2021). For medical image segmentation tasks, considering the insufficient feature map recovery and lack of spatial consistency of FCNs, Shoeibi et al. (2024) proposed a variant network of FCN called U-Net. Understanding of the brain networks that control appetite is developing quickly thanks to developments in neuro-technology for mapping, modifying, and tracking molecularly defined cell types. Here, we go over these crucial instruments and how they are used in the circuits that regulate the desire and intake of food. These tools’ technical capabilities create a strict experimental framework for studying the neuroscience of hunger. This network consists of symmetric encoders and decoders, using skip connections to concatenate down sampled and up sampled features to retain more dimensional and spatial information (Stampfl et al., 2023). BTS was one of several medical picture segmentation tasks that it swiftly became the standard approach for, because to its streamlined network design and outstanding segmentation performance (Zhao and Zhao, 2021). Combining up- and down-sampled features in order to preserve more spatial and dimensional information. Up sampling is the process of increasing the rate of an already sampled signal, and down sampling is the process of decreasing the rate. Up sampling is the process of altering a signal to erroneously raise the sampling rate. Considering the limitations of 2D U-Net in capturing contextual information in 3D MRI brain tumor images, researchers proposed 3DU-Net (Vanderbecq et al., 2020), significantly improving the segmentation performance of 2D network models (Li et al., 2023). In addition, addressing issues such as insufficient high-resolution feature representation of small-scale and irregular brain tumor regions based on 3DU-Net (Smith et al., 2023), researchers introduced autoencoders (Yousefirizi et al., 2022), attention mechanisms (Zhao et al., 2023), and cascade architectures into the network (Niyas et al., 2022), encouraging research into 3DU-Net models for BTS (Venkatesan et al., 2022). Myronenko (Li et al., 2023) improved the accuracy of BTS by cascading a VAE into the 3DU-Net on the cascade network for BTS. When it came time for the 2018 BTS Challenge (BraTS), this approach took first place. Researchers suggested 3DU-Net, which greatly enhances the segmentation performance of 2D network models, in response to the shortcomings of 2D U-Net in capturing contextual information in 3D MRI brain tumor pictures. Since the 2D CNN starts with ImageNet weights, it converges more quickly than the 3D CNN, whose weights are initialized at random.

Subsequently, Smith et al. (2023) and Yousefirizi et al. (2022) used cascade 3DU-Nets to segment brain tumours, obtaining rough predictive segmentation results in the first stage and combining the rough segmentation results with 3D brain tumor MRI images in the subsequent phase to foretell outcomes of finer segmentation. Smith et al. (2023) method won first place in the BraTS 2019 Challenge. Furthermore, similarly designed multi-stage cascade 3DU-Net methods from coarse to fine and fully utilized multi-scale information. The techniques clearly show that cascade networks perform better in BTS, which makes it a significant area of study in this area. This study is motivated by the remarkable performance of a 3D cascaded network in BTS tests. to solve the drawbacks of U-Net networks, including their small local receptive fields, loss of geographical information, and underuse of contextual information. A 3D cascaded network’s exceptional performance in BTS tests is the basis for this study. To address the shortcomings of U-Net networks, such as their limited local receptive fields, spatial information loss, and contextual information underutilization, this work builds on 3D cascaded networks to improve the network’s ability to express global feature attention and adaptively adjust its receptive fields. U-NET is a popular option in many medical imaging applications due to its unique properties, which are often used for its accuracy in picture segmentation. A decoding path known as the expanding path and an encoding path known as the contracting path are combined in U-NET:

- A novel 3D cascaded BTS network design is presented, based on the existing one.
- To enhance the network’s ability to capture features over long distances, ICU-Net presents a non-local expectation maximization attention (EMA) module that is lightweight.
- The efficiency of the suggested method is demonstrated by both ablation and comparative experimental findings, which indicate competitive performance in comparison to the field’s most advanced techniques.

Firstly, to enhance the global context information of brain tumours and expand the receptive field, a lightweight non-local EMA module (Cervantes et al., 2023) is introduced into the cascaded network. Additionally, to address the difficulty of capturing tumour features of different shapes and sizes effectively with regular convolutions, an attention-based dynamic convolution (Dutande et al., 2021) is attempted in the network. Neuronal morphology gene expression (Lein et al., 2007), markers of excitatory or inhibitory neurotransmitter release, and electrophysiological firing properties are among the utilitarian criteria that have been used to define cell types and are significant to different groups of experimenters (Jennings et al., 2013). The development and mature transcription factor codes information theory stimulus response sensitivity, axon projection targets along with intersections of these criteria (Dasen and Jessell, 2009). The field of cough analysis has received little attention from AI experts. Several things might be blamed for this, such as inefficient auxiliary frameworks, expensive database acquisition, or difficulties developing classifiers (Kumar et al., 2021). A lightweight non-local EMA module is added to the cascaded network in order to improve the global context information of brain tumours and increase the receptive field. Additionally, an attention-based dynamic convolution is tried in the network to solve the challenge of efficiently capturing tumour features of various shapes and sizes using normal convolutions. This dynamic convolution adapts its kernel based on input dynamically, better accommodating the significant differences in brain tumor images, thereby further improving network performance without deepening or widening the network.

In this paper, loss functions are used in two stages. The output is calculated using the cross-entropy loss function in the network’s initial stage in comparison to the ground truth image. The ground truth image is compared using the cross-entropy loss function and the dice loss function.

In summary, this article constructs a new model for BTS, named Cascade Dynamic Attention U-Net (ICU-Net), which integrates EMA and dynamic convolution into a 3D cascaded U-Net framework. The main contributions are as follows:

1. Based on the 3D cascaded BTS network architecture, a new 3D cascaded BTS network is proposed, focusing on enhancing the network’s ability to express tumor global context information and adaptively adjust the receptive field for tumours of different scales.
2. In the fourth connection of the second-stage network, ICU-Net introduces a lightweight non-local EMA module to improve long-distance feature capture by the network. Simultaneously, all regular convolutions in the second-stage network are replaced with dynamic convolutions to adaptively match local feature receptive fields, achieving overall improvement in BTS performance.
3. ICU-Net is validated on the BraTS 2019 and BraTS 2020 datasets. Both ablation and comparative experimental results demonstrate the effectiveness of the proposed method, showing competitive performance compared to state-of-the-art methods in the field.

Neurotechnology is profitable to make new connections between previously disconnected neural system components very soon. This has the potential to significantly improve the lives of those with autism, anxiety, and chronic pain. There are very few ways to change the stimulation of the nervous system and very little continuous sensing.

The paper is organized into 4 sections, initially, Section 1 provides Introduction section; Section 2 covers Network Framework and Algorithm Principles; Section 3 presents Experiment and Results the major conclusions drawn from the study in the Section 4.

## 2 Network framework and algorithm principles

### 2.1 Complete network framework

**Figure 1:**
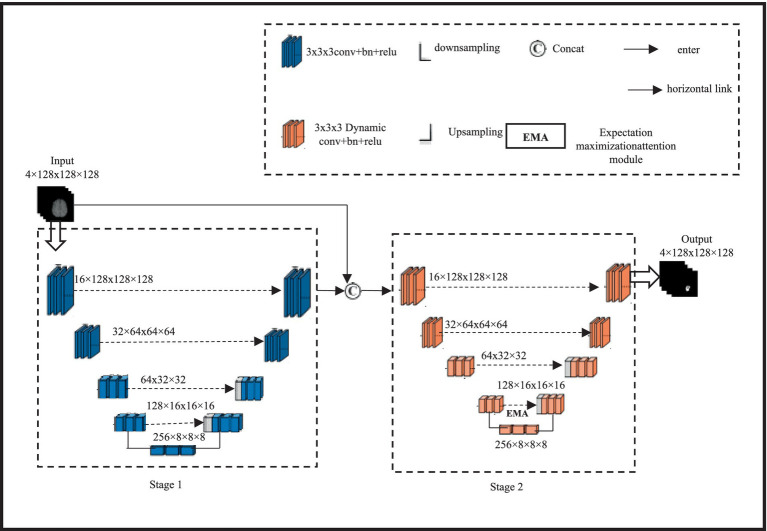
BTS network architecture based on cascaded dynamic attention 3D U-Net design and spatial information of brain tumours.

The proposed 3D cascaded dynamic attention U-Net BTS network architecture is shown in Figure 1.

A two-stage network is formed by cascading two 3DU-Net networks with four layers of up sampling and down sampling; this architecture achieves segmentation results for brain tumor regions ranging from coarse to fine, allowing for the reconstruction of finer high-resolution spatial information. Initially, a 3DU-Net is employed to roughly segment brain tumours; the input size for this multi-modal tumor is 4 × 128 × 128 × 128 and the output size is left unchanged. In the second stage, the input of the network is the aggregation of the rough segmentation result and the multi-modal 3D brain tumor MRI, with a size of 8 × 128 × 128 × 128. By learning aggregated information, the rough segmentation result guides the network to learn finer segmentation results from 3D brain tumor MRI. The final output size remains 4 × 128 × 128 × 128. EMA module is presented to capture long-distance feature dependencies in characterizing enhancing tumour (ET) images. The EMA module is friendly to memory and computation, and it is resistant to input variance. In addition, we established the normalization and maintenance procedures to sustain the training process. Due to the limitation of using conventional direct connections in the lateral connections of 3DU-Net, which fails to capture long-distance feature dependencies in describing enhancing tumor (ET) images, a lightweight non-local EMA module is introduced. A small, non-local EMA module to enhance the network’s ability to capture long-range features. The second-stage network simultaneously replaces all of its conventional convolutions with dynamic convolutions to adaptively fit local feature receptive fields and improve overall BTS performance. Considering cost estimation using computational intelligence, the EMA module is only added at the fourth lateral connection of the second stage. The input feature size of this module is 128 × 16 × 16 × 16, expanding the network’s receptive field while enhancing global information representation. Additionally, all regular convolutions in the encoding and decoding modules of the second-stage 3DU-Net are replaced with dynamic convolutions to adaptively adjust the network’s receptive field based on tumor size, thereby strengthening the network’s ability to extract local tumor features.

#### 2.1.1 Expectation maximization attention

**Figure 2:**
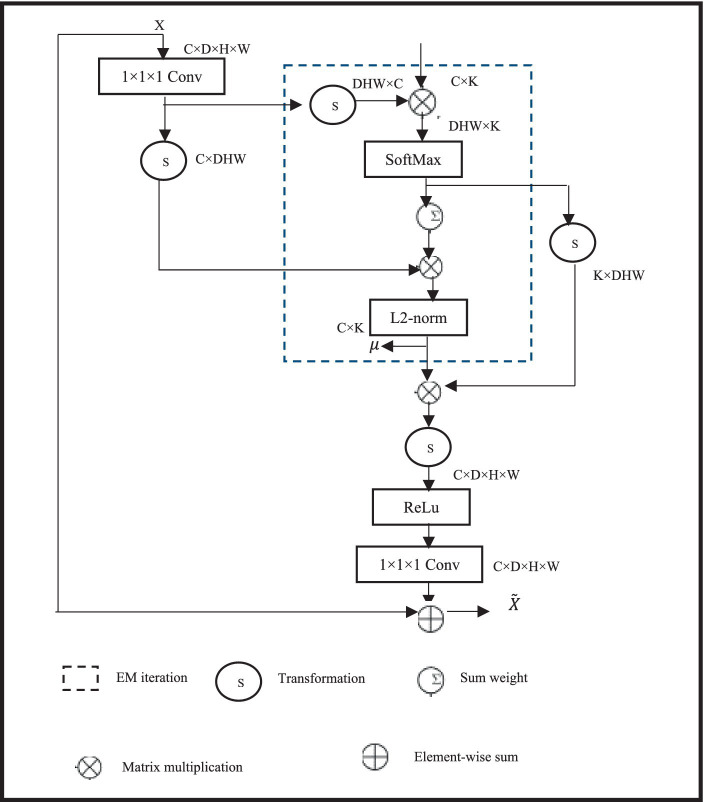
Structure of the expectation maximization attention module.

Global information is crucial for BTS, and introducing non-local self-attention can enhance long-distance feature dependencies in Wholetumor (WT) images. However, conventional non-local self-attention requires point-wise calculation of spatial attention and generates attention maps, leading to a sharp increase in model cost estimation using computational intelligence when combined with 3D convolutions. Therefore, a lightweight non-local EMA stage is used, which calculates worldwide focus via means of reconstructed features. Since the number of voxels in the reconstruction base is much smaller than that of the input feature map, the cost estimation using computational intelligence of non-local attention can be significantly reduced. Early diagnosis improves the prognosis and prospects of a successful course of treatment for brain tumours. Early detection can also lessen the chance of consequences for the patients and lessen the damage to the surrounding healthy brain tissue. An early diagnosis of brain tumours is essential for bettering the prognosis and outcomes for patients. By identifying the warning signs and symptoms and getting help right away, people can guarantee a rapid diagnosis and have access to a variety of treatment choices. The specific structure of the EMA module is shown in Figure 2. It uses 1 × 1 × 1 convolution to change the channel number of the feature map X, and inputs it into the EM algorithm inside the dashed box to calculate non-local attention. To avoid overfitting, the input feature map is summed with the reconstructed feature map in a residual manner.

For the specific optimization calculation of the module, the EMA module uses the Expectation Maximization (EM) algorithm to iterate a compact set of bases, and then calculates attention on this set of bases. Given input feature map X ∈S^D × E × G × Y^ and initial bases μ ∈S^D × K^, where D is the number of channels, E × G × Y is the size of the input feature map, and K is the number of bases, the bases μ and non-local self-attention A∈S^E × G × Y × K^ learn parameters and latent variables, respectively. The goal of the EM algorithm is to find the optimal learnable parameters to maximize the data likelihood. The EM process mainly involves Expectation (E) step and Maximization (M) step. The F step estimates the expectation of A, while the M step updates through likelihood maximization. The specific process is as follows in Eqs. (1, 2):

(1) $$A^{\left(u\right)}=\mathrm{sofUmax}\left(X^{U}\left(\mu^{\left(u-1\right)}\right)\right)$$

(2) $$\mu_{k}^{\left(u\right)}=\frac{\sum_{n=1}^{w}\left(A_{nk}^{\left(u\right)}X_{n}\right)}{\sum_{n=1}^{w}A_{nk}^{\left(u\right)}}$$

Where, t represents the number of iterations, W = E × Y × G, and A_nk_ is the attention vector of the k-th channel at position n. In this study, t is set to 3, meaning that the F step and M step are alternately executed three times. Finally, the reconstructed feature map $\tilde{X}$ is obtained through the converged μ and normalized attention A, represented in Eq. (3) as follows:

(3) $$\tilde{X}=\mu A^{U}$$

Incorporating the EMA module into the network effectively aggregates long-distance contextual information and reduces intra-class differences while preserving inter-class differences, thereby enhancing the segmentation performance of the cascaded BTS network.

#### 2.1.2 Dynamic convolution

In deep neural networks, when using regular convolutions, after the network training is completed, all convolutional kernel parameters are fixed, and the convolution shares the convolutional kernel parameters. To improve performance, more parameters must be learned through network deepening or expanding. Through the use of the idea of dynamic convolution, which enables the convolutional layer to adaptively alter its receptive field based to the properties of the input picture segmentation, the model performance may be improved without expanding the depth or width of the network. In order to improve the model performance without increasing the depth or width of the network, Ye et al. (2021) and Yasin et al. (2024) proposed the concept of dynamic convolution, allowing the convolutional layer to adaptively adjust its receptive field according to the characteristics of the input image segmentation.

**Figure 3:**
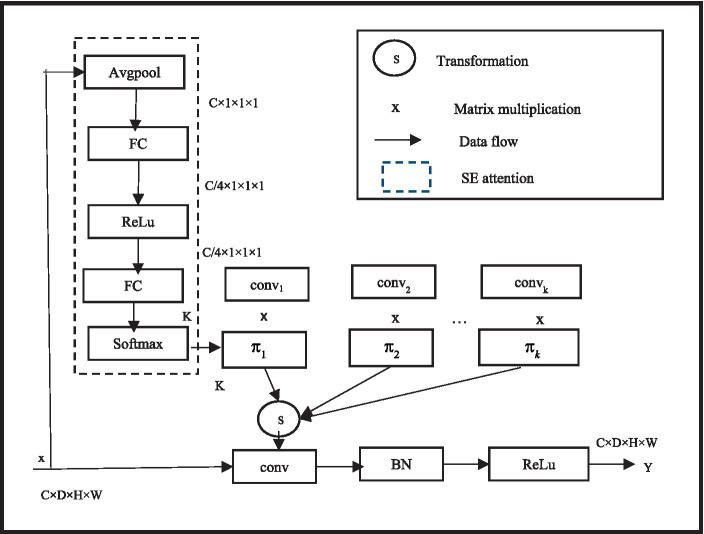
Dynamic convolution structure diagram.

In specific implementation, dynamic convolution uses a set of K parallel convolution kernels instead of using one convolution kernel per layer. The dynamical convolutional and EMA module in detail later in this article. In addition, the outputs of the first and second stages are independently compared with the ground truth pictures to compute loss values, which are then combined into a final loss value to jointly supervise the network in order to improve network training. Based on the focus of the input picture segmentation, the convolution kernels dynamically aggregate numerous parallel convolution kernels and aggregate them in a non-linear manner to achieve greater feature representation capacity. In the meantime, dynamic convolution computes the attention of convolution kernels using the squeeze and excitation module SENet. By compressing the global spatial information of the input through global average pooling, and then reducing the dimensionality of the features using two fully connected layers and an intermediate non-linear layer structure. Among them, the first fully connected layer reduces the dimensionality, the second fully connected layer reduces the dimensionality to K, and finally softmax generates normalized attention weights π k for K convolution kernels. The three-dimensional MRI brain tumor images are the result of this study’s extension of the two-dimensional dynamic convolution to three dimensions. Moreover, three-dimensional dynamic convolutions are used in place of all normal convolutions in the second stage of the cascaded network, allowing the network to adaptively determine convolution kernel parameters for various input brain tumor images and improve BTS outcomes. To facilitate the learning of attention $\pi_{k}$ , $\pi_{k}$ is constrained to $\underset{k}{\sum }\pi_{k}\left(x\right)=1$ . Unlike SENet, dynamic convolution applies attention to parallel convolutions, so the additional attention calculation cost is very low. The specific structure of dynamic convolution is shown in Figure 3.

Since MRI brain tumor images are three-dimensional, this study extends the original two-dimensional dynamic convolution to three-dimensional. Although attention layers are based on human concepts of attention, they are just a weighted mean reduction. The query, the values, and the keys are the three inputs that the attention layer receives. When the query consists of a single key and the keys and values are equivalent, these inputs are frequently similar. Furthermore, all regular convolutions in the second stage of the cascaded network are replaced with three-dimensional dynamic convolutions, enabling the network to adaptively establish convolution kernel parameters for different input brain tumor images, thus achieving better BTS results. The convolutional neural network’s overall training pace and prediction capacity are somewhat influenced by the parameters of each layer. Determining the ideal parameter configuration is therefore a crucial step in convolutional neural network training.

### 2.2 Loss function

The loss function is an important factor guiding the training of the BTS network. Considering the severe class imbalance problem in BTS tasks, the cross-entropy loss function L_CE_ and the Dice loss function L_DC_ are combined in the loss function. The cross-entropy loss reduces the error between the predicted result and the ground truth image, while the Dice loss function, commonly used in medical image segmentation, effectively addresses the class imbalance problem. Their formulas are shown in in Eqs. (4, 5) respectively as:

(4) $$L_{CE}=-\overset{M}{\underset{i=0}{\sum }}x_{i}\log{\hat{x}}_{i}$$

(5) $$L_{DC}=1-\frac{2\sum_{i=0}^{M}x_{i}{\hat{x}}_{i}}{\sum_{i=0}^{M}\left(x_{i}+{\hat{x}}_{i}\right)}$$

Where *x* represents the ground truth image, $\hat{x\;}$ represents the predicted segmentation result, and M is the total number of sample labels.

This paper divides the use of loss functions into two stages. In the first stage of the network, the output is computed with the cross-entropy loss function against the ground truth image. The BTS network’s training is significantly influenced by the loss function. The cross-entropy loss function (LCE) and the dice loss function (LDC) are used in the loss function to address the serious class imbalance issue in BTS tasks. The Dice loss function, which is frequently used in medical picture segmentation, successfully addresses the class imbalance issue, while the cross-entropy loss minimizes the error between the predicted result and the ground truth image. In the second stage, the combination of cross-entropy loss function and Dice loss function is used against the ground truth image. Subsequently, the two-stage loss functions are added together proportionally to obtain the complete Loss function, represented in Eq. (6) as:

(6) $$\mathrm{Loss}=0.5\times L_{CE1}+0.5\times \left(L_{CE2}+L_{DC2}\right)$$

$L_{CE2}\;\mathrm{and}\;L_{DC2}$ respectively represent the cross-entropy loss functions of the first and second stages of the BTS network, and LDC2 represents the Dice loss function of the second stage of the network.

## 3 Experiment and results

### 3.1 Experimental environment and configuration

The experimental setup includes a 3.80GHz Intel(R) Xeon(R) Gold 5,222 CPU and a 24GB Nvidia RTX 3090 GPU. Python and the Adam optimizer are used for both code implementation and model training. A weight decay coefficient of 1 × 10^–5^ should be used, momentum of 0.95, and an initial learning rate of 0.001. Due to the 3D BTS model’s high memory consumption and hardware constraints, the batch size during training is 3 and the model training iterations are 550. Utilizing cascaded 3D U-Net and 3D U-Net networks, the Brain Tumour Segmentation Challenge 2019 dataset (BRATS 2019) enabled the automatic segmentation of brain tumours in magnetic resonance imaging (MRI) images. The brain tumor segmentation is first broken down into three segments: the enhanced tumour (ET), the tumour core (TC), and the entire tumour (WT).

### 3.2 Dataset and pre-processing

**Figure 4:**
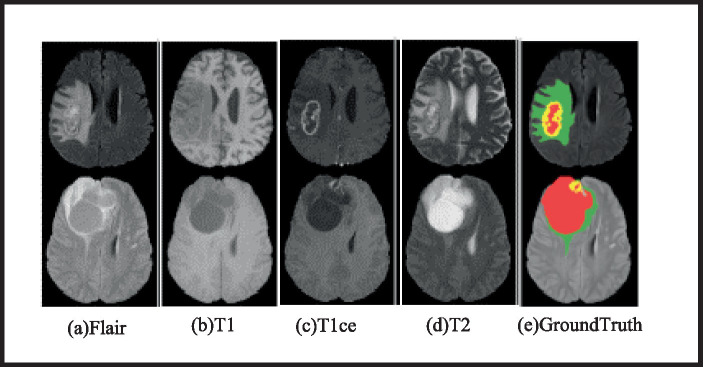
MRI brain tumor visuals with four modalities and ground truth.

To test the approach, BraTS 2019 and BraTS 2020, public MRI BTS datasets, are used. BraTS 2019 has 335 training and 125 validation cases. The training set includes 259 HGG and 76 LGG instances. BraTS 2020 improves the training set by 369 HGG instances and maintains the validation set at 125. The BraTS 2020 training set comprises 293 HGG and 76 LGG. Four modes per case: Flair, U1, U2, and U1ce, and each MR image’s size is 240 mm × 240 mm × 155 mm. Additionally, the training set provides manually segmented brain tumor results by professional physicians. The training set offers manually segmented results of brain tumours by medical professionals. The validation set does not reveal ground truth picture labels in order to maintain fairness in BTS results. Additionally, segmentation results must be submitted to an online evaluation portal in order for model performance to be assessed. Four classifications of brain tumor imaging data labels are healthy (0), necrotic (1), edoema (2), and enhancing (4). WT, TC, and ET lesion locations are classified. Each tumor lesion class—WT, TC, and ET—has a label. Figure 4 shows two example MRI brain tumor imaging samples: an HGG sample and an LGG sample. Red indicates necrotic areas, green edoema regions, and yellow enhancing tumours.

In the pre-processing of brain tumor image data, the A-score method is initially used for dataset standardization, as shown in Eq. 7.

(7) $$A^{\prime }=\frac{A-\mu }{\delta }$$

Where, A stand for the input picture, A′ for the normalised image, μ represents the average, while δ represents the norm of deviation.

The network is a 4 × 128 × 128 × 128 resized version of the multi-modal three-dimensional brain tumor MRI pictures, which are cropped at random since brain tumor images include a substantial quantity of useless background information. Furthermore, techniques for enhancing picture data are employed, including random rotation, flipping, and intensity adjustments.

### 3.3 Evaluation metrics

To measure the model’s performance, we employ the widely-used Dice Similarity Coefficient (DSC) and housekeeping distance. For segmentation evaluation, the empirical discrepancy methods have been the most widely utilized techniques. By comparing the segmented image with a manually segmented reference image—often referred to as the ground truth and calculating error measurements, these techniques assess segmentation techniques. To measure how close the segmentation output is to the ground truth picture, a measure that can take on values between zero and one, the Dice Similarity Coefficient is computed in this way:

(8) $$\mathrm{Dicescore}=\frac{2UQ}{HQ+2UQ+HN}$$

UQ, HQ, UN, and HN denote the number of tumor voxels successfully predicted, non-tumor voxels predicted, and tumor voxels not identified.

To evaluate the distance between the segmentation result boundary and the ground truth boundary, use the Hausdorff distance formula: where u and q represent points in the ground truth and predicted regions, respectively, and *d*(*t*, *p*) is the distance function between points *t* and *p*. Hausdorff95 multiplies the final Hausdorff value by 95% to remove outliers.

### 3.4 Experiments and results

First, we perform ablation experiments using the BraTS 2020 dataset to confirm the efficiency of the proposed ICU-Net BTS model. Then, we compare it to other comparable networks in the field. Additionally, experimental results comparing ICU-Net to other models on the BraTS 2019 dataset are presented to demonstrate its generalisation performance. Lastly, the efficiency of the suggested methodology is further shown by presenting visual segmentation data.

#### 3.4.1 Ablation experiment results

Both the datasets used for training and validating BraTS 2020 were used to conduct the experiments to completely validate the outcomes of the ablation. As a starting point, we utilised the 3DU-Net and then transformed it into ainto a 3D transmitted U-Net using EMA and dynamic convolution modules in the second stage. This allowed us to validate the results more effectively. For simplicity’s sake, we’ll refer to the cascaded U-Net model as CU-Net; “+EMA” is for the inclusion of Expectation Maximisation Attention, and “+DConv” stands for Dynamic Convolution. The pictures of brain tumours in this dataset were randomly divided into two sets—one for training and one for validation—in a ratio of 80:20 for the BraTS 2020 ablation studies. Additionally, to account for training duration and result stability, we trained each model twice and averaged the results. You may see the detailed outcomes of the ablation tests in Table 1.

**Table 1:** Ablation experiments on the BraTS 2020 training dataset.

Segmentation method	DSC			
	ET	WT	TC	Average
U-Net (baseline method)	0.784	0.9	0.797	0.827
CU-Net	0.788	0.897	0.816	0.834
CU-Net + EMA	0.803	0.898	0.815	0.839
CU-Net + DConv	0.797	0.905	0.84	0.847
ICU-Net	0.813	0.905	0.836	0.851

Table 1 shows that the baseline model, 3DU-Net, achieved DSC values of 0.784 for ET, 0.900 for WT, and 0.797 forTC. With the fundamental cascaded model, CU-Net, these values changed to 0.788, 0.897, and 0.816, respectively. In comparison to the baseline model, the WT result was somewhat lower, but the ET and TC outcomes were better. Particularly, there was a 1.9% accuracy improvement in TC, verifying the effectiveness of the cascaded architecture. Furthermore, when the EMA module or dynamic convolution module was individually added to the cascaded model, there were overall performance improvements compared to the basic cascaded method. Adding the EMA module improved ET by 1.5% points, while adding dynamic convolution improved ET by 0.9% points and TC by 2.4% points, showing that these two modules are successful in small-scale BTS. The cascaded design was completed by including both modules to create ICU-Ne. The resulting DSC values for ET, WT, and TC were 0.813, 0.905, and 0.836, respectively. These represented performance improvements of 2.5, 0.5, and 2% over the cascaded basic model CU-Net, and 2.9, 0.5, and 3.9% over the baseline U-Net method, indicating the good performance of BTS using the ICU-Ne model. This is since the added modules can better capture tumor global context information and adaptively adjust the sensitivity to tumor regions. Meninges, pituitary gland, craniopharyngeal duct, and frontal and temporal lobes are the most often affected brain regions in adults. Most typically, the cerebellum and brainstem are the sites of brain tumours in children. In medical image analysis, automated high-precision BTS is currently a challenging task due of the irregular and ill-defined borders, varying sizes, and shapes of brain tumours.

The BraTS 2020 validation set was used for additional ablation experiments to verify the results. We trained the models using all the BraTS 2020 training data, then used them to segment 125 brain tumours in the validation set. Finally, the BraTS online assessment platform returned the index evaluation findings from the segmentation results. Table 2 lists experimental outcomes.

**Table 2:** Validation data from BraTS 2020 for ablation trials.

Segmentation method	DSC			
	ET	WT	TC	Average
U-Net (baseline method)	0.769	0.894	0.799	0.821
CU-Net	0.773	0.897	0.808	0.826
CU-Net + EMA	0.787	0.899	0.815	0.834
CU-Net + DConv	0.778	0.895	0.833	0.835
ICU-Net	0.786	0.903	0.828	0.839

Table 2 shows that the 3DU-Net baseline technique obtained DSC findings of 0.769 for ET, 0.894 for WT, and 0.799 for TC. Each of these parameters saw an improvement of 0.4 percent, 0.3 percent, and 0.9% following network cascading. Adding the EMA module and dynamic convolution separately to the cascaded network improved BTS by 1.8 and 0.9% in ET and 1.6 and 3.4% in TC, respectively. This contrasted with the small difference in the WT metric when compared to the 3DU-Net. In conclusion, ICU-Net achieved ET performance of 0.786, WT performance of 0.903, and TC performance of 0.828 after incorporating the cascade and EMA modules and dynamic convolution. These results demonstrated that the cascaded design in conjunction with the two modules was effective, as they showed gains of 1.7, 0.9, and 2.9% when compared to 3DU-Net. Furthermore, the table data shows that overall performance was ideal across all three criteria, even if individual metrics were not satisfied in the results.

#### 3.4.2 Comparison with representative methods

Tables 3, 4 compare the proposed BTS approach against others using the BraTS 2019 and BraTS 2020 validation sets to prove its efficacy and competitiveness. On the BraTS 2019 validation set, ICU-Net averaged 0.834 DSC values for ET, WT, and TC, which were 0.781, 0.897, and 0.826, respectively (Table 3). With average DSC values of 0.799 and 0.788, Ahamed et al. (2023) improved the loss function of a 3DU-Net++ network and Wu et al. (2023) built a unique BTS model utilising cross-stage local network design. Cannet presented a context-guided attention conditional random field using high-dimensional feature maps (Stampfl et al., 2023). ICU-Net outperforms author techniques. As compared to Liu et al., ICU-Net increased ET, WT, and TC by 2.2, 1.2, and 2.5% while outperforming CANet. This method outperformed multi-stage cascaded networks by 3.3 and 1.1%, respectively, confirming its usefulness. In TransBTS (Niyas et al., 2022), the Transformer, a popular computer vision tool, was used to segment 3D brain tumours. Both methods yielded similar averages. Averaging 5.69 Hausdorff distance, the ICU-Net technique ranks third in the table, with ET, WT, and TC values of 3.39, 5.91, and 7.77. This paper’s DSC and Hausdorff distance results proved competitive with other representative approaches.

**Table 3:** Comparison results with representative methods the BraTS2019 testing dataset.

Segmentation method	DSC				Hausdorff95			
	ET	WT	TC	Average	ET	WT	TC	Average
Ahamed et al. (2023)	0.709	0.873	0.814	0.799	12.3	15.45	12.47	13.4
Wu et al. (2023)	0.707	0.878	0.779	0.788	–	–	–	–
Stampfl et al. (2023)	0.759	0.885	0.851	0.832	4.8	5.89	6.56	5.75
Yousefirizi et al. (2022)	0.75	0.854	0.8	0.801	3.08	7.04	5.96	5.36
Zhao et al. (2023)	0.771	0.886	0.813	0.823	6.03	6.23	7.41	6.55
Lundervold and Lundervold (2019)	0.789	0.9	0.819	0.836	3.73	5.64	6.04	5.14
ICU-Net	0.781	0.897	0.826	0.834	3.39	5.91	7.77	5.69

**Table 4:** Comparison results with representative methods on the BraTS 2020 validation dataset.

Segmentation method	DSC				Hausdorff95			
	ET	WT	TC	Average	ET	WT	TC	Average
Zhao and Zhao (2021)	0.729	0.886	0.802	0.806	31.97	10.26	13.58	18.6
Vanderbecq et al. (2020)	0.77	0.896	0.839	0.835	32.4	7.7	11.7	17.26
Niyas et al. (2022)	0.764	0.882	0.801	0.816	21.39	6.49	6.68	11.52
Shoeibi et al. (2024)	0.79	0.897	0.829	0.838	24.14	6.17	7.04	12.45
Newsome (2006)	0.774	0.891	0.803	0.823	26.84	15.78	8.56	17.06
ICU-Net	0.786	0.903	0.828	0.839	35.03	4.57	14.58	18.06

Table 4 displays BraTS 2020 validate set findings, Newsome (2006) proposed an improved 3DU-Net by using dilated convolutions (Erus et al., 2020) and adding attention in skip connections (Boveiri et al., 2020), which showed significant performance advantages compared to the original 3DU-Net (Bui et al., 2019). Bernal (2002) added an EMA module to the lateral connections of the DMF-Net model (Bao et al., 2003). Compared to this, ICU-Net outperformed by 1.6% on ET but lagged by 1.1% on TC, with an overall performance improvement of 0.4% on average. In comparison with cascaded networks, Salvoro et al. (2017) proposed a segmentation network with three sub-branches and one main branch (Seifert, 1987), where the sub-branches captured different brain tumor features (Kondhalkar and Parab, 2013), and the main branch aggregated multi-modal features using spatial-channel fusion blocks (Liu et al., 2023). Singh et al. (2021) used a two-stage VAE cascaded network and added attention gates in the network (Eppinger et al., 2021). Compared to author and author cascaded BTS networks, the ICU-Net method outperformed author by 2.3% and author by 0.1% on average. Additionally, compared to Ienca and Ignatiadis (2020) SwinBTS method, which added Swin Transformer to the 3DU-Net, ICU-Net led by 1.2, 1.2, and 2.5% on ET, WT, and TC, respectively. These results show that the suggested BTS approach works. The ICU-Net technique obtained 4.57 on WT but significantly less on ET and TC in Hausdorff distance. This is because ICU-Net did not use more post-processing, which may lead to inaccurate predictions for individual LGGs without ET, causing false positives and significantly affecting the model evaluation results.

#### 3.4.3 Results visualization

**Figure 5:**
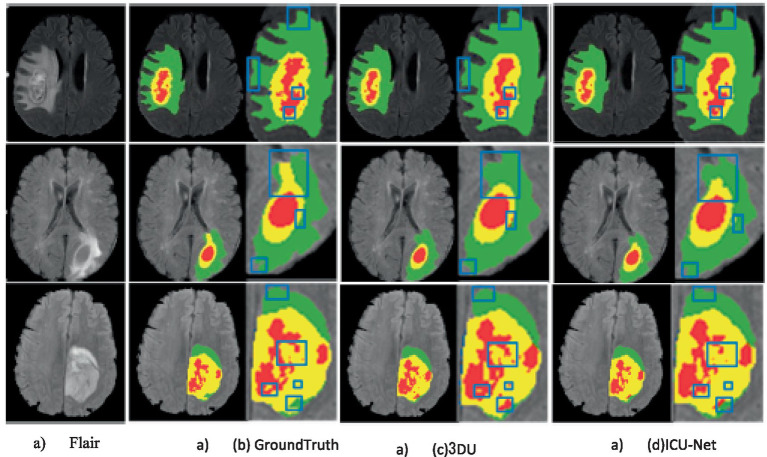
Example of segmentation results on the BraTS 2020 training set (color images are available in the electronic version).

To visually display the BTS results more intuitively, the results of the BraTS 2020 train datasets were visualized, as display in Figure 5. Three representative cases were selected for display in Figure 5, with each row demonstrating the Flair image, Ground Truth, 3DU-Net, and Left to right, we can see the results of the ICU-Net segmentation. On top of the Flair picture are the segmentation results from 3DU-Net, ICU-Net, and Ground Truth. Additionally, to highlight the segmentation results where ICU-Net outperforms U-Net, blue lines are used. Through the visualized images, it can be observed that the proposed method can effectively segment enhancing tumours, WT, and tumor cores, outperforming the 3DU-Net. However, there is still some gap compared to the Ground Truth, indicating room for further improvement in segmentation performance.

## 4 Conclusion

The introduction of the EMA module and Dynamic Convolution has effectively enhanced the network’s ability to capture both global and local tumor information. The results of the ablation experiments on the publicly available BraTS 2019 and BraTS 2020 datasets demonstrate the effectiveness of the introduced modules in BTS. Moreover, the comparison results with representative methods in the field further prove that the suggested method is competitive. In future work, to further enhance the accuracy of BTS, we will explore cascaded architectures using convolutional and self-attention modules that have better feature expression capabilities. Additionally, we will study more sophisticated data augmentation tactics and post-processing approaches. The EMA module will be covered in the future. In addition, the outputs of the first and second stages are independently compared with the ground truth pictures to compute loss values, which are then combined into a final loss value to jointly supervise the network in order to improve network training.

## Data availability statement

Publicly available datasets were analyzed in this study. This data can be found at: https://www.kaggle.com/datasets/aryashah2k/brain-tumor-segmentation-brats-2019, https://www.kaggle.com/datasets/awsaf49/brats2020-training-data.

## Author contributions

HB: Conceptualization, Data curation, Methodology, Visualization, Writing – original draft. MA-K: Conceptualization, Data curation, Resources, Visualization, Writing – original draft. AD: Conceptualization, Methodology, Resources, Validation, Visualization, Writing – original draft. FA: Methodology, Resources, Software, Visualization, Writing – original draft. MS: Investigation, Methodology, Project administration, Supervision, Visualization, Writing – review & editing. MB: Methodology, Software, Validation, Visualization, Writing – review & editing. VC: Data curation, Formal analysis, Investigation, Validation, Writing – review & editing. KS: Formal analysis, Funding acquisition, Investigation, Validation, Writing – review & editing. RJ: Formal analysis, Resources, Validation, Writing – original draft, Writing – review & editing.
